# Management of serious autolytic attempt in the Emergency Room

**DOI:** 10.1192/j.eurpsy.2025.2351

**Published:** 2025-08-26

**Authors:** Y. D’Hiver Cantalejo, M. F. Tascón Guerra, T. Jamed Muñoz, A. Osca Oliver

**Affiliations:** 1 Psiquiatria, Hospital Nuestra Señora Del Prado, Talavera de la Reina, Spain

## Abstract

**Introduction:**

Every year, 726,000 people take their own lives and many more attempt it. Suicides can occur at any age and were the third most common cause of death in people aged 15 to 29 worldwide in 2021.

**Objectives:**

Presentation of a clinical case.

**Methods:**

We analyze the case of a 17-year-old patient who came to the ED after ingesting sodium hypochlorite with self-lytic intent. She says that, being accompanied by a friend, she begins to hear “a voice, which is my own voice, telling me to kill myself.” With a pretext, he enters the kitchen and overeats. She says that, although she was induced by “the voice,” she thinks that “if I continue like this all my life, it would be better to die.” She discusses it with her brother and her friend, who inform her mother.

She is the youngest of three brothers. He resides with his mother and her partner, parents divorced at 11 years old. He is in 4th ESO, with poor performance. Pregnancy, childbirth and maturation milestones within normality. Four years ago he began to experience behavioral alterations in the family environment characterized by drug abuse reactive to family arguments. These ingestions are becoming more frequent and for anxiolytic purposes, requiring attention in the ED. Throughout evolution, the attitude has become increasingly regressive, with demands for attention to which the family responds by reinforcing them. He has had several hospital admissions. On current treatment with olanzapine 5 mg/24h, fluoxetine 20 mg/24h and tranxilium 5 mg/8h.

**Results:**

Analysis with blood count, basic biochemistry, arterial blood gases, SO and toxic substances in urine; without significant alterations.

Gastroscopy: Esophagus: Mucosa, distensibility and peristalsis without alterations. Esophago-gastric junction 36 cm from the dental arch with competent cardia at the level. Stomach: isolated antral areas of circumscribed erythema. Centered and permeable pylorus. Duodenum: Bulb and second portion without alterations.

Psychopathological examination: COC. Regressive, character traits in the foreground. No alterations in psychomotor skills. Attentive, without memory errors. Discourse with an infantilized tone, spontaneous, fluid and coherent, structured, focused on feelings of vital failure. Referred hypothymia, without apathy or hypohedonia. Referred anxiety, not evidenced. Active autolytic ideation, without criticism, manifesting intentionality of repetition. Low tolerance for frustration with impulsive responses. Preserved appetite. Hypersomnia. Preserved reality judgment. Partial awareness of illness.

**Conclusions:**

Suicidal behavior should never be considered a call for attention but rather for help. In the intervention we must not blame and reconnect the minor with the family. We must talk openly about the circumstances in which it occurred, facilitating emotional expression. We must guarantee the safety of the minor, open dialogue between parent-child and provide support from parents.

**Disclosure of Interest:**

None Declared

